# Pressure Anesthesia

**Published:** 1899-09

**Authors:** Wm. J. Morton


ARTICLE II.

### PRESSURE ANESTHESIA.

DR. WM. J. MORTON.

Mr. President and Gentlemen of the Odontological Society:—I need not say how deeply I appreciate the honor of having the pleasure of being with you this evening and taking part in these discussions, which tend in the end to ameliorate the hard duties of professional life such as both you and I have to meet every day.

Think of the charming goal of which we are in pursuit—to relieve the patient of pain. Then think of the equally agreeable effect upon the operator, to relieve himself of the strain of seeing the patient suffer pain, for I am sure you must feel the pain.

Now there is a humorous side to obtaining any new principle in medicine, just as in anything else. I heard a dentist remark a few minutes ago that since these new things came up, if you will excuse my levity in repeating what he said, it had played "the deuce" with his patients. I suppose he meant he had been experimenting upon them.

Now, my doctrine is something like the doctrine of an acute observer who noticed that when two cats began disputing on his roof, instead of annihilating each other, as he hoped they would, more cats came on the roof. Now that is the effect I would like to see from this new subject. I want to see more cats come on the roof, and I want to hear some other person say that he also has experimented.

I believe that out of all this agitation and thinking there will come in the end some simple, easy means of producing anesthesia of sensitive dentin, which we are now particularly interested in, that will be so easy that you wonder why you have not always done it.

I think we are in what might be called an agitatory stage of the subject. And no matter how old I get, I want to retain that young enthusiasm that wants a try at each new thing, I want to see obtunding, whether by cataphoric anesthesia or any other means, practiced until we are all settled as to which is a best and easiest way.

To-night we are to have, I understand, simply an informal conversation; I yielded to the extremely persuasive powers of one of your members, Dr. Walker, who overcame all my objections, but it was on the understanding that you would do a great deal more of the talking than I. Some of what I shall say is, after all, in a certain respect retrospective, as well as antrospective; if there be such a word. There is a little historical reminiscence back of pressure anesthesia. What, for instance, do we mean by pressure anesthesia? Simply that something exerts a pressure—some obtunding or anesthetizing agency is forced or finds its way into those dentinal tubules and there produces anesthesia. Cataphoresis was pressure anesthesia in an electrical sense, for electricity is pressure, and that is all it is. Cataphoresis, so called, using the positive pole of the battery to exert an electric pressure, was therefore pressure anesthesia. That we will not refer to to-night. Cataphoresis is very much alive. No alternative procedure of so simple a nature can die out of practice, for cataphoresis *will* produce numbness of sensitive dentin, and there will always be cases where you can employ it, but it is also a fact that a simpler method would be desirable. This simpler process we are to-night in search of.

When, four years ago, I stood before the meeting of the First District Society, and we went over the ground of cataphoresis from A to Z, it was a difficult subject because new—difficult to receive and grasp—and, moreover, there are always certain technical difficulties that will stand around cataphoreses, but at this day great advances in the practice have been made.

If, however, possibly there is some simpler method in say twenty per cent. of the cases, we ought to have it in view, and by and by one method will be put against the other until we settle down to some fixed practice.

I wrote a little book on cataphoresis in which I went over the ground fully, and in that book I put one short chapter, just a page and a half, entitled "Pressure Anesthesia." That was an anchor thrown out to the windward about two years ago. Fond as I was of cataphoresis, there still clung in my mind this idea, that simple mechanical pressure, or the pressure of gases and vapors, might finally be used to convey anesthetizing material into the substance of the dentin, and so I put in that chapter.

This idea I had also brought forward at a meeting in the White Mountains in July, 1897, on the subject of pressure anesthesia. The discussion was published in *Items of Interest.*

With your permission, I will read briefly a part of the chapter above referred to.

"*Pressure Local Anesthesia of Soft Tissues and of Dentin.*"—It has become a well-established practice in dental surgery to introduce into the cavity of a tooth for the purpose of bleaching it, when the cavity admits of it, a twenty five or other convenient per cent. of peroxid of hydrogen, held on a pledget of cotton and sealed in. It is maintained that, in addition to the simple process of inhibition, the cocain is forced into the substance of the dentin by pressure due to the evaporation of the ether. In this manner teeth can be successfully bleached to an extent not possible if the pledget of cotton holding the bleaching fluid is not sealed in, except when electricity is used. It occurred to me that if this same principle could be applied to a sulfuric ether solution of cocain that we should possess an extraordinarily simple method of anesthetizing the soft tissues in general and sensitive dentin in particular—a method so simple and sure that it would sweep away all the labor that has been expended in a certain class of cavities, upon the introduction of cocain by electricity. Since cocain hydro-chlorate would not dissolve in sulfuric ether, I dissolved it in guaiacol (making a ten per cent. solution), and then found that I could add equal parts of sulfuric ether to the solution without precipitating the cocain.

"Experiment 1. June 23rd, 1897.—Taking about twenty drops of the above solution, I put it in a small test tube, and inverting the tube, I held its mouth firmly against the skin of my forearm. At the end of five minutes, removing the tube, I tested the area of skin comprised within the circle represented by the mouth of the tube, and found that it was perfectly anesthetized; it remained anesthetic for five minutes, and then gradually recovered sensation without any hyperesthesia to speak of and without injury to the skin. Further experiments substantiated this result.

"I have also made some experiments with air pressure by connecting to a rubber tube, itself in connection with a cylinder of compressed air and a gauge, a hollow needle having a shoulder near its extremity. Where the cavity permits of it, the solution of cocain is introduced upon absorbent material sealed in with plastic material, and then the needle thrust through it as far as the shoulder will permit. At this point the air pressure is allowed to act. By the use of this instrument local anesthesia is promptly obtained. Dr. M. L. Rhein has now an instrument of this description in his possession, and will as soon as possible make further experiments upon dentin and report some cases."

These two publications placed this question of pressure anesthesia fairly before the dental profession two years ago. Since that time I have given very little thought to the matter. I had reports from some of the gentlemen very early. I had a report from Dr. Van Woert, of Brooklyn, who wrote to me he had made several experiments, and perhaps we will hear from him later on where he used these experiments.

Beyond a few more such reports, I hardly knew what had come from that idea, until within the last few months I observe that the profession has become largely interested in pressure anesthesia produced by ethereal solutions of cocain as I had suggested and demonstrated. If on account of its odor it is not desired to use guaiacol as an intermediary to get the otherwise insoluble cocain salt into solution with ether, any other intermediary, such as, for instance, alcohol, may be employed. How the solution is effected is a minor question, once the fact having been established that it could be done and that the solution would act.

What, then, I propose to present for consideration to-night is new to our profession—to make practical applications of *continuous* air pressure for driving any substance through the dentinal tubuli, so that the substance mixes in and through and, being medicinal, like cocain, produces an anesthetizing effect. This same view would be true of pressure if you object that the air would not find its way easily enough through these tubules. Hydrogen will find way; nitrous oxid would find its way under pressure. In fact, you have the whole realm of gases as well as air. But I will confine myself entirely to air, because I have experimented mostly with it.

I am first going to pass around this bulb. You see it is the usual one used for the Pacquelin cautery, for a Davidson syringe; in short, the ordinary one used in dentistry, which you blow up and produce a continual pressure. To its tube is attached a little ebonite cup about one inch in diameter and one-eighth of an inch deep. This is not for dental use, but I show it by way of illustration. Suppose that I wish to take guaiacol and cocain to anesthetize the skin of the arm. I take a ten or twenty per cent. solution of hydrochlorate of cocain in guaiacol, and if the skin is to be anesthetized, smear it upon the arm, place this little cup against it, and blow this bulb up, and hold or tie it there. You have a continued elastic pressure going on here; you can hear it escaping here as I raise the edge; there is quite a pressure. It is a question of pressure versus pressure. There is the blood pressure on the one hand, and the air pressure on the other. If the air pressure is greater than the blood pressure, then of course the blood is going to retreat into the mass of the skin and the anesthetizing solution will follow it, and you get an anesthetized spot which you can prick and cut without pain. This I have demonstrated beyond question in actual practice.

Now we will see how to apply this to dental practice. Let us illustrate on the blackboard. We have a source of air pressure, a rubber bulb, a cylinder like a bicycle pump or an air pump—any source of air pressure you like. We have our anesthetizing agent; this is a solution of cocain in sulfuric ether, though other solutions may be found to act equally well or better. But the ether solution seems to have a special advantage, and I think it is because the sulfuric ether holds the cocain and finds its way down into the tubles, evaporates, leaves the cocain, and the cocain then takes up a partnership with the fluid lymph of the tubule. The sulfuric ether is what enables the cocain to join in with the lymph of the dentinal tubuli. This also I conceive to be the action of the guaiacol when this is made use of with cocain.

We must also take into account the devitalizing effect of pressure upon the sensitive structure of the tooth; but there is another important truth to consider. As soon as you leave the arterial system and come down to the capillary system, which we represent here by "C" [illustrating by means of a diagram plotted to scale], there is at least one-fifth fall in the blood pressure. In the capillary system this blood pressure comes about to equalize the pressure of the venous system. The actual blood pressure then, when it comes to the capillary system, is very small. The walls of the capillaries have no particular strength, and the blood circulates about in them under very little pressure.

When, therefore, the arterial pressure reaches the pulp-cavity of the tooth it is practically a capillary pressure. That means that you have got very little resistance to overcome by means of your artificial pressure, and this is just what is wanted.

I make here a very rough outline of a tooth which has a cavity in it. For convenience, we will only represent three dentinal tubuli. There is your capillary pressure in there [illustrating on blackboard] at the end of the line. I am going to set up against it an air pressure. I will pass around various teeth that I secured to-day, with cavities in them, fitted with pieces of rubber tubing. The tubing is small, thin and highly elastic, such as all are familar with. Pass the end of a rubber tube like that over the crown of the tooth, and ligature it tight with a bit of floss silk around the neck of the tooth. Then attach the other end of this tubing to your source of air pressure. At this point you have a little stopcock. Apply the pressure and allow it to act continuously.

If you set up and say an ounce pressure, that ounce of pressure is exercised; the cavity contains the anesthetizing material upon absorbent cotton; put on pressure with the air bulb, and that pressure is translated into a movement of anesthetizing material into the tubules.

The air bulb now acts continuosly by virtue of its elasticity, and you ask the patient to sit down ten, fifteen or twenty minutes, and a continuous effect is being produced by driving the substance into this sensative dentin. We all know how simple the principle is of hydraulic pressure. A very small tube, carrying a jet of water flowing a long time, will exert an enormous ultimate force.

In the case of the instrument I passed around, the familiar pair of rubber bulbs, about five minutes was enough to drive the anesthetizing agent into the skin. In the case of sensitive dentin it might require from that to fifteen minutes.

In fact, the whole process is so utterly simple that it requires very little explanation; it seems that all we have to deal with here is the internal blood pressure—namely, the capillary pressure within the tooth, and the forcing into the sensitive dentin of fluids by reason of the artificial pressure.

I have here a diagram which illustrates what I have said and which I will pass around. I have gone over the ground a little hurriedly, but the method in its simplicity speaks for itself.

Unlike cataphoresis, where there is foundation after foundation to be laid, here in this pressure anesthesia we are treading upon very familiar ground, and if it should prove to be practical in a certain number of cases we should be making a very considerable advance.

I myself believe that practice will lead to several procedures very simple and easy, and that we have got to be familiar with these several procedures.

Now, if you use a sulfuric ether solution of the hydrochlorate of cocain without sealing it with rubber cement, as I in the beginning advised, if you use it without sealing it in or without putting up against it any instrument to retain the ether vapor, then you have still, to a certain extent, a pressure anesthesia by reason of the packing, but not exactly in the sense that I first meant it.

What I present new to-night, and beyond the record, is the simple fact represented by this tooth which I have passed around—a tooth upon which has been passed a rubber tube, which in turn is connected with a rubber bulb, maintaining a continuous air pressure. Anesthetizing material is placed in contact with the sensitive dentin of the tooth thus arranged.

I am sure that enough has been said to open and pave the way for further discussion to-night, and I hope we shall hear from some of those who have had experience in this direction. I can only hope that this simple procedure may appeal to your practical sense, and that it may add to and enhance the value of a great number of experiments that I personally know are taking place in your profession in this matter. Probably more of your number are experimenting in this direction than you have any idea of. At this point I will close what I have to say with an apology for my very elemental presentation of the subject.—*Cosmos.*

